# Subtype-Independent Activation of NF-κB Signaling in Breast Cancer

**DOI:** 10.3390/ijms27094055

**Published:** 2026-04-30

**Authors:** Elżbieta Mitka-Krysiak, Katarzyna Król-Jatręga, Piotr Ossowski, Nikola Zmarzły, Krzysztof Bereza, Paweł Ordon, Tomasz Sirek, Agata Sirek, Kacper Boroń, Dariusz Boroń, Grzegorz Wyrobiec, Tomasz Szczepanik, Marta Skorek, Beniamin Oskar Grabarek

**Affiliations:** 1 Collegium Medicum, WSB University, 41-300 Dabrowa Gornicza, Poland; katarzynakroljatrenga@gmail.com (K.K.-J.); drpiotrossowski15@gmail.com (P.O.); nikola.zmarzly@gmail.com (N.Z.); pawelordon4@gmail.com (P.O.); drtstierka@gmail.com (T.S.); agatasirek06@gmail.com (A.S.); dariusz@boron.pl (D.B.); tomekdok@o2.pl (T.S.); mskorek@wsb.edu.pl (M.S.); bgrabarek7@gmail.com (B.O.G.); 2 Department of Mother and Child Health, Faculty of Health Sciences, Institute of Nursing and Midwifery, Jagiellonian University Medical College, 31-008 Kraków, Poland; kbereza@su.krakow.pl; 3 Department of Plastic Surgery, Faculty of Medicine, Academia of Silesia, 40-555 Katowice, Poland; q374@gmail.com; 4 Department of Plastic and Reconstructive Surgery, Hospital for Minimally Invasive and Reconstructive Surgery in Bielsko-Biała, 43-316 Bielsko-Biala, Poland; 5 Faculty of Medicine and Health Sciences, Andrzej Frycz Modrzewski University in Kraków, 30-705 Kraków, Poland; 6 Department of Gynecology and Obstetrics, TOMMED Specjalisci od Zdrowia, 40-055 Katowice, Poland; 7 Department of Gynecology and Obstetrics with Gynecologic Oncology, Ludwik Rydygier Memorial Specialized Hospital, 31-826 Kraków, Poland; 8 Faculty of Medicine, VIZJA University, 01-043 Warsaw, Poland; 9 Department of Histology and Cell Pathology in Zabrze, Faculty of Medical Sciences in Zabrze, Medical University of Silesia in Katowice, 41-808 Zabrze, Poland; gwyrobiec@sum.edu.pl

**Keywords:** breast cancer, micro RNA (miRNA), nuclear factor kappa B (NF-κB) signaling

## Abstract

Nuclear factor kappa B (NF-κB) signaling plays a central role in inflammation, immunity, cell survival, and cancer progression. Its constitutive activation is frequently observed in breast cancer, contributing to tumor growth, treatment resistance, and metastasis. MicroRNAs (miRNAs) are key post-transcriptional regulators of gene expression and may modulate NF-κB signaling in a subtype-specific or -independent manner. The aim of the study was to identify miRNAs that may potentially regulate the activity of genes associated with NF-κB signaling across five molecular subtypes of breast cancer in Polish women. Tumor and matched normal tissue samples were collected from 405 patients with five breast cancer subtypes: luminal A (*n* = 130), HER2-negative luminal B (*n* = 100), HER2-positive luminal B (*n* = 96), non-luminal HER2-positive (*n* = 36), and triple-negative breast cancer (TNBC, *n* = 43). Expression profile of selected NF-κB-related genes were evaluated using mRNA microarrays and RT-qPCR. Protein levels were assessed by ELISA. Candidate regulatory miRNAs were identified via miRNA microarrays and validated using the miRDB database. A consistent upregulation of *MAP3K7*, *TAB2*, *TNFAIP3*, *CSNK2A1*, *BCL2L1*, *XIAP*, *CXCL2*, and *PLAU* was observed across all subtypes, suggesting activation of canonical NF-κB signaling. Downregulation of specific miRNAs, miR-1297 and miR-30a (targeting *MAP3K7*), miR-134 (*TAB2*), miR-125b (*TNFAIP3*), and miR-4329 (*XIAP*), may contribute to this deregulation. For *CSNK2A1*, *BCL2L1*, *CXCL2*, and *PLAU*, no regulatory miRNAs meeting our criteria were identified. Our study reveals a subtype-independent activation of the canonical NF-κB signaling pathway in breast cancer, underpinned by consistent upregulation of key components (at both the transcript and protein levels. Dysregulation of specific miRNAs likely contributes to this altered gene expression. These findings suggest the presence of a common NF-κB-driven oncogenic program across molecular subtypes, with potential implications for developing miRNA-based therapeutic strategies targeting inflammation, survival signaling, and treatment resistance in breast cancer.

## 1. Introduction

In women worldwide, breast cancer remains the most prevalent malignancy and a leading cause of oncological mortality [1]. Recent data from the Polish National Cancer Registry reveal that in 2022, it ranked second among cancer-related deaths in women, with a disproportionately high impact on women under 45 [2]. Molecular classification of breast cancer, based on ER, PR, and HER2 status, defines biologically distinct subtypes with varying proliferative potential, prognosis, and response to therapy. Notably, more aggressive subtypes such as triple-negative and HER2-positive tumors often exhibit enhanced inflammatory signaling [3,4,5].

Nuclear factor kappa B (NF-κB) signaling pathway is a central regulatory axis involved in immune response, inflammation, cell proliferation, and survival [6]. In the canonical signaling, stimulation by pro-inflammatory cytokines activates the IκB kinase (IKK) complex, which phosphorylates inhibitor of κB alpha (IκBα), marking it for ubiquitination and subsequent proteasomal degradation. This degradation frees NF-κB dimers, typically composed of p50 and p65 (RELA), allowing their translocation to the nucleus, where they regulate the transcription of a wide range of target genes. In contrast, the non-canonical pathway does not depend on IκBα degradation but instead relies on the stabilization and activation of NF-κB-inducing kinase (NIK), which leads to the processing of p100 into p52 and the formation of p52-RELB dimers that translocate to the nucleus [7].

Aberrant or constitutive activation of NF-κB has been widely documented in various malignancies, including breast cancer [8]. It is associated with key oncogenic processes such as increased cell proliferation, resistance to apoptosis, epithelial–mesenchymal transition (EMT), angiogenesis, and increased metastatic potential [9,10,11]. In addition, NF-κB is involved in the remodeling of the tumor microenvironment by inducing pro-inflammatory mediators and immune modulators, thus contributing to tumor progression and treatment resistance [12]. The NF-κB pathway is subject to multilayered and complex regulation, the outcome of which may depend on the stimulus, context, or cell type [13,14]. In recent years, increasing attention has been paid to the role of noncoding RNAs, particularly microRNAs (miRNAs), in modulating NF-κB signaling [15,16,17,18,19]. MiRNAs can act as positive or negative regulators of this pathway by targeting mRNAs encoding its components or related regulatory proteins [20]. Given the complexity and context-dependent nature of NF-κB action, elucidating its interactions with miRNAs may shed new light on the molecular basis of breast cancer pathogenesis as well as identifying potential targets for therapeutic intervention.

The aim of the study was to identify miRNAs that may potentially regulate the expression of genes associated with NF-κB signaling in five subtypes of breast cancer in Polish women.

## 2. Results

### 2.1. Gene Expression Profile Determined with mRNA Microarrays

**Figure 1:**
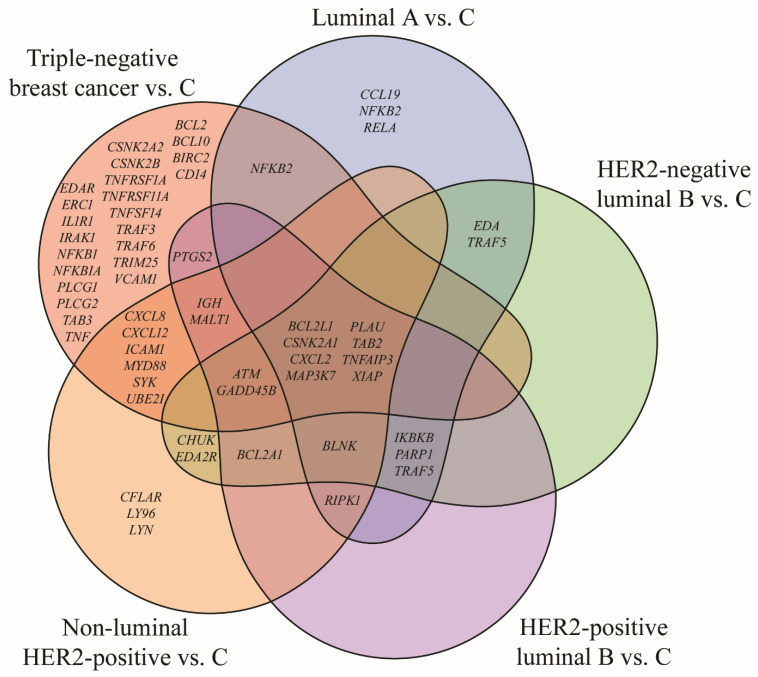
Venn diagram of genes involved in the NF-κB signaling pathway differentiating breast cancer from the control (*p* < 0.05; FC > 2 or <−2). HER2, human epidermal growth factor receptor 2; C, control; *ATM*, ataxia telangiectasia mutated; *BCL2*, B-cell lymphoma 2; *BCL2A1*, BCL2 related protein A1; *BCL2L1*, BCL2 like 1; *BCL10*, B-cell lymphoma/leukemia 10; *BIRC2*, baculoviral IAP repeat containing 2; *BLNK*, B-cell linker; *CCL19*, CC-motif chemokine ligand 19; *CD14*, cluster of differentiation 14; *CFLAR*, CASP8 and FADD-like apoptosis regulator; *CHUK*, conserved helix–loop–helix ubiquitous kinase; *CSNK2A1*, casein kinase 2 alpha 1; *CSNK2A2*, casein kinase 2 alpha 2; *CSNK2B*, casein kinase 2 beta; *CXCL2*, C-X-C motif chemokine ligand 2; *CXCL8*, C-X-C motif chemokine ligand 8; *CXCL12*, C-X-C motif chemokine ligand 12; *EDA*, ectodysplasin A; *EDA2R*, ectodysplasin A2 receptor; *EDAR*, ectodysplasin receptor; *ERC1*, ELKS/RAB6-interacting/CAST family member 1; *GADD45B*, growth arrest and DNA-damage-inducible beta; *ICAM1*, intercellular adhesion molecule 1; *IGH*, immunoglobulin heavy chain; *IKBKB*, inhibitor of nuclear factor kappa B kinase subunit beta; *IL1R1*, interleukin 1 receptor type 1; *IRAK1*, interleukin-1 receptor-associated kinase 1; *LY96*, lymphocyte antigen 96; *LYN*, LYN proto-oncogene Src family tyrosine kinase; *MALT1*, mucosa-associated lymphoid tissue lymphoma translocation 1 gene; *MAP3K7*, mitogen-activated protein kinase kinase kinase 7; *MYD88*, myeloid differentiation primary response 88; *NFKB1*, nuclear factor kappa B subunit 1; *NFKB2*, nuclear factor kappa B subunit 2; *NFKBIA*, NFKB inhibitor alpha; *PARP1*, poly (ADP-ribose) polymerase 1; *PLAU*, urokinase; *PLCG1*, phospholipase C gamma 1; *PLCG2*, phospholipase C gamma 2; *RELA*, nuclear factor kappa B p65 subunit; *RIPK1*, receptor interacting serine/threonine kinase 1; *SYK*, spleen associated tyrosine kinase; *TAB2*, TGF-beta activated kinase 1 (*MAP3K7*) binding protein 2; *TAB3*, TGF-beta activated kinase 1 (*MAP3K7*) binding protein 3; *TNF*, tumor necrosis factor; *TNFAIP3*, tumor necrosis factor alpha-induced protein 3; *TNFRSF1A*, tumor necrosis factor receptor superfamily member 1A; *TNFRSF11A*, tumor necrosis factor receptor superfamily member 11A; *TNFSF14*, tumor necrosis factor superfamily member 14; *TRAF3*, TNF receptor-associated factor 3; *TRAF5*, TNF receptor-associated factor 5; *TRAF6*, TNF receptor-associated factor 6; *TRIM25*, tripartite motif-containing 25; *UBE2I*, ubiquitin conjugating enzyme E2 I; *VCAM1*, vascular cell adhesion molecule 1; *XIAP*, X-linked inhibitor of apoptosis protein.

Out of 260 mRNAs corresponding to 105 NF-κB pathway-related genes, 86 mRNAs were found to be significantly dysregulated in tumor samples compared to adjacent non-cancerous tissue (one-way ANOVA, *p* < 0.05; FC > 2 or <−2). Subtype-specific analysis using Tukey’s post hoc test identified significant expression changes in 27 mRNAs for the luminal A group, 29 for HER2-negative luminal B, 29 for HER2-positive luminal B, 43 for non-luminal HER2-positive, and 69 for triple-negative breast cancer (TNBC). Figure 1 displays a Venn diagram illustrating the distribution of subtype-specific and overlapping gene alterations.

No uniquely altered genes were identified for the HER2-negative or HER2-positive luminal B subtypes. Changes in *CCL19*, *NFKB2* and *RELA* expression were characteristic for luminal A subtype, while *CFLAR*, *LY96* and *LYN* expression changes were characteristic for non-luminal HER2-positive subtype. The highest number of characteristic genes, i.e., 23, was noted for TNBC. In addition, eight genes showed consistent differential expression across all breast cancer subtypes when compared to control tissue: *BCL2LI*, *CSNK2A1*, *CXCL2*, *MAP3K7*, *PLAU*, *TAB2*, *TNFAIP3*, *XIAP* (Table 1).

Consistent across all breast cancer subtypes, a statistically significant upregulation was observed for *BCL2L1*, *CSNK2A1*, *CXCL2*, *MAP3K7*, *PLAU*, *TAB2*, *TNFAIP3*, *XIAP*.

### 2.2. Expression Profile of BCL2L1, CSNK2A1, CXCL2, MAP3K7, PLAU, TAB2, TNFAIP3, XIAP Determined with RT-qPCR and ELISA

**Figure 2:**
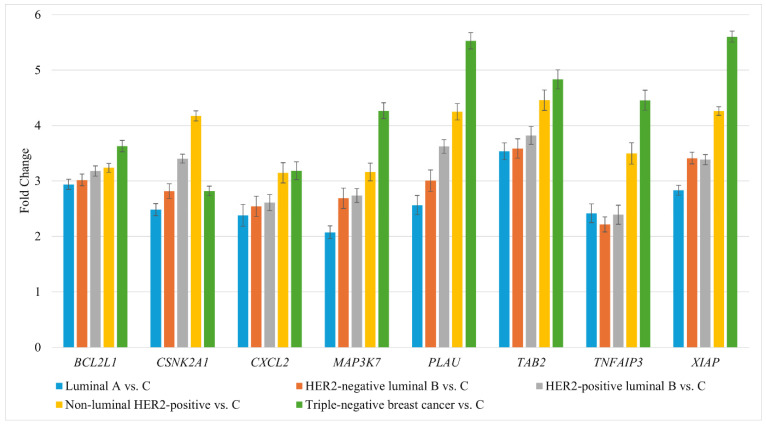
RT-qPCR-based expression profiles of *BCL2L1*, *CSNK2A1*, *CXCL2*, *MAP3K7*, *PLAU*, *TAB2*, *TNFAIP3*, and *XIAP*, showing consistent dysregulation across all breast cancer subtypes compared to the control. HER2, human epidermal growth factor receptor 2; C, control; *BCL2L1*, BCL2 like 1; *CSNK2A1*, casein kinase 2 alpha 1; *CXCL2*, C-X-C motif chemokine ligand 2; *MAP3K7*, mitogen-activated protein kinase kinase kinase 7; *PLAU*, urokinase; *TAB2*, TGF-beta activated kinase 1 (*MAP3K7*) binding protein 2; *TNFAIP3*, tumor necrosis factor alpha-induced protein 3; *XIAP*, X-linked inhibitor of apoptosis protein. Data are presented as mean ± standard deviation.

RT-qPCR was used to validate the expression levels of *BCL2L1*, *CSNK2A1*, *CXCL2*, *MAP3K7*, *PLAU*, *TAB2*, *TNFAIP3*, *XIAP* (Figure 2).

The RT-qPCR results were consistent with the gene expression patterns obtained from the microarray analysis. Subsequently, protein levels of the selected genes were quantified using ELISA (Table 2).

The level of *BCL2L1*, *CSNK2A1*, *CXCL2*, *MAP3K7*, *PLAU*, *TAB2*, *TNFAIP3*, *XIAP* was significantly increased in all tumor samples compared to the control group. This was consistent with the microarray and RT-qPCR results.

### 2.3. miRNA Target Prediction

The next step was to verify whether *BCL2L1*, *CSNK2A1*, *CXCL2*, *MAP3K7*, *PLAU*, *TAB2*, *TNFAIP3*, *XIAP* could be targets of miRNAs differentiating breast cancer from the control (Table 3).

The analysis showed that miRNAs identified by microarrays and prediction criteria did not target *BCL2L1*, *CSNK2A1*, *CXCL2*, or *PLAU*. Overexpression of *MAP3K7* may be associated with reduced levels of miR-1297 and miR-30a. In addition, overexpression of *TAB2*, *TNFAIP3*, and *XIAP* may be a consequence of decreased levels of miR-134, miR-125b, and miR-4329, respectively.

Importantly, all identified miRNA–mRNA pairs demonstrated inverse expression patterns, characterized by downregulation of miRNAs and upregulation of their predicted target genes. This relationship is consistent with canonical miRNA-mediated repression and suggests a loss of post-transcriptional regulatory control contributing to increased expression of NF-κB-related genes.

To increase the reliability of predicted interactions, miRNA–mRNA pairs identified using the miRDB database were further cross-validated using the TargetScan database. This analysis confirmed the majority of predicted regulatory relationships, including miR-30a/*MAP3K7*, miR-134/*TAB2*, miR-125b/*TNFAIP3*, and miR-4329/*XIAP*. In contrast, the interaction between miR-1297 and *MAP3K7* was not supported by TargetScan, suggesting that this particular pair should be interpreted with greater caution.

**Figure 3:**
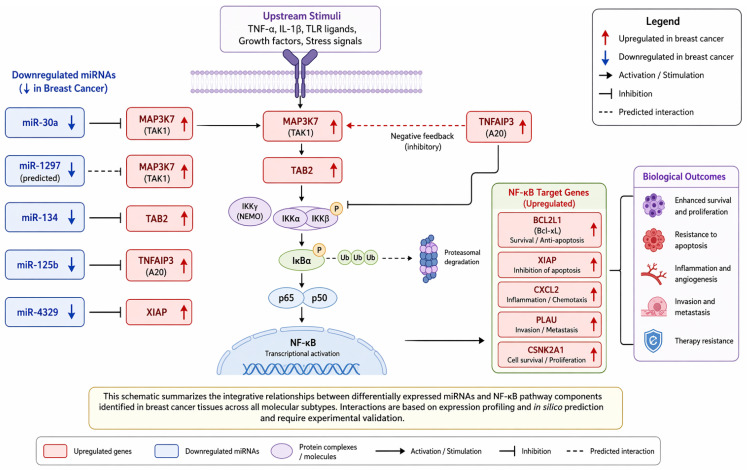
Schematic representation of miRNA–mRNA interactions within NF-κB signaling in breast cancer.

To facilitate interpretation of the identified miRNA–mRNA interactions and their relationship to NF-κB signaling, we constructed an integrative schematic model summarizing the regulatory network observed in this study (Figure 3).

The schematic summarizes the relationships between differentially expressed miRNAs and NF-κB pathway components identified across all molecular subtypes of breast cancer. Upregulated genes (red) include *MAP3K7*, *TAB2*, *TNFAIP3*, *XIAP*, *BCL2L1*, *CSNK2A1*, *CXCL2*, and *PLAU*, while downregulated miRNAs (blue) include miR-30a, miR-1297, miR-134, miR-125b, and miR-4329. Arrows indicate activation within the NF-κB signaling cascade, whereas blunt-ended lines represent inhibitory miRNA–mRNA interactions. Dashed lines indicate predicted interactions requiring further validation. The network reflects expression-based associations supported by target prediction databases and does not represent direct functional evidence of pathway activation.

### 2.4. Overall Survival Analysis

**Figure 4:**
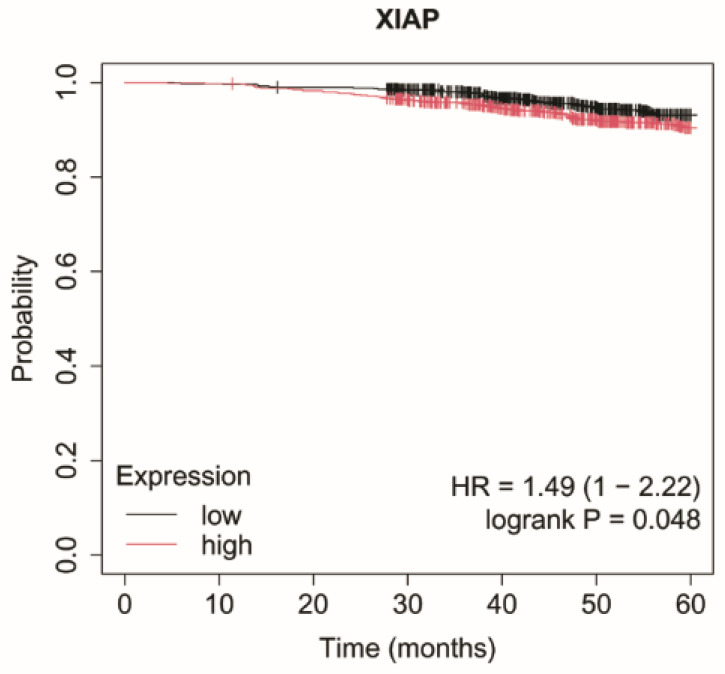
Kaplan–Meier overall survival curves for the luminal A subtype based on *XIAP* expression. Data were obtained from the Kaplan–Meier plotter database (http://kmplot.com/; accessed: 17 June 2025). Patients were stratified into high- and low-expression groups using the median expression value. Survival differences were evaluated using the log-rank test. Censored observations are indicated by tick marks. The follow-up period was limited to 60 months. *XIAP*, X-linked inhibitor of apoptosis protein.

**Figure 5:**
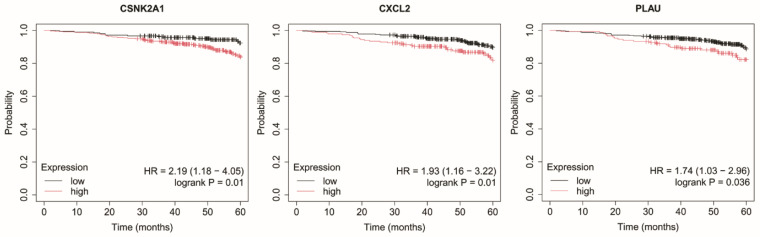
Kaplan–Meier overall survival curves for the HER2-negative luminal B subtype based on *CSNK2A1*, *CXCL2*, and *PLAU* expression. Data were obtained from the Kaplan–Meier plotter database (http://kmplot.com/; accessed: 17 June 2025). Patients were stratified into high- and low-expression groups using the median expression value. Survival differences were evaluated using the log-rank test. Censored observations are indicated by tick marks. The follow-up period was limited to 60 months. *CSNK2A1*, casein kinase 2 alpha 1; *CXCL2*, C-X-C motif chemokine ligand 2; *PLAU*, urokinase.

**Figure 6:**
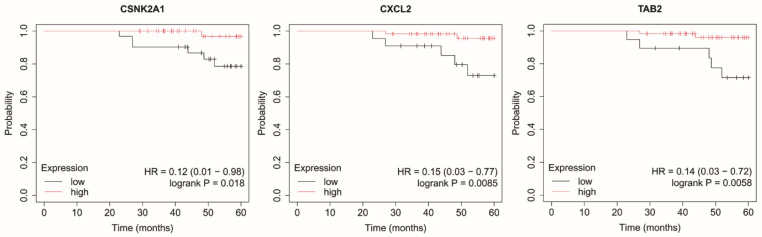
Kaplan–Meier overall survival curves for the HER2-positive luminal B subtype based on *CSNK2A1*, *CXCL2*, and *TAB2* expression. Data were obtained from the Kaplan–Meier plotter database (http://kmplot.com/; accessed: 17 June 2025). Patients were stratified into high- and low-expression groups using the median expression value. Survival differences were evaluated using the log-rank test. Censored observations are indicated by tick marks. The follow-up period was limited to 60 months. *CSNK2A1*, casein kinase 2 alpha 1; *CXCL2*, C-X-C motif chemokine ligand 2; *TAB2*, TGF-beta activated kinase 1 (*MAP3K7*) binding protein 2.

**Figure 7:**
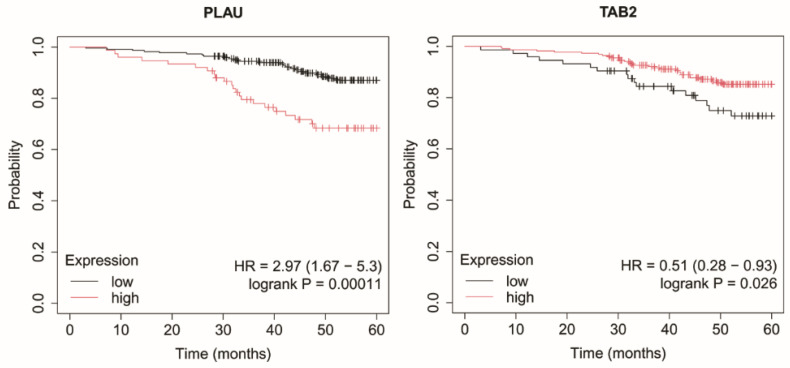
Kaplan–Meier overall survival curves for the non-luminal HER2-positive subtype based on *PLAU* and *TAB2* expression. Data were obtained from the Kaplan–Meier plotter database (http://kmplot.com/; accessed: 17 June 2025). Patients were stratified into high- and low-expression groups using the median expression value. Survival differences were evaluated using the log-rank test. Censored observations are indicated by tick marks. The follow-up period was limited to 60 months. *PLAU*, urokinase; *TAB2*, TGF-beta activated kinase 1 (*MAP3K7*) binding protein 2.

**Figure 8:**
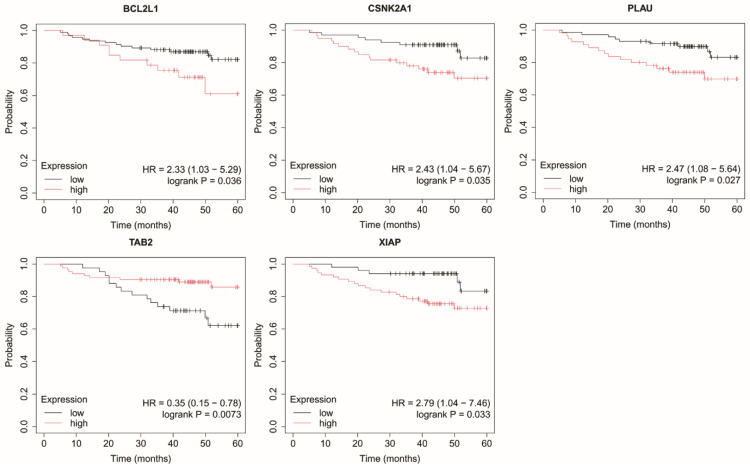
Kaplan–Meier overall survival curves for triple-negative breast cancer based on *BCL2L1*, *CSNK2A1*, *PLAU*, *TAB2*, and *XIAP* expression. Data were obtained from the Kaplan–Meier plotter database (http://kmplot.com/; accessed: 17 June 2025). Patients were stratified into high- and low-expression groups using the median expression value. Survival differences were evaluated using the log-rank test. Censored observations are indicated by tick marks. The follow-up period was limited to 60 months. *BCL2L1*, BCL2 like 1; *CSNK2A1*, casein kinase 2 alpha 1; *PLAU*, urokinase; *TAB2*, TGF-beta activated kinase 1 (*MAP3K7*) binding protein 2; *XIAP*, X-linked inhibitor of apoptosis protein.

Overall survival (OS) analysis was performed for *BCL2L1*, *CSNK2A1*, *CXCL2*, *MAP3K7*, *PLAU*, *TAB2*, *TNFAIP3*, *XIAP*. For each breast cancer subtype, only survival curves with statistically significant associations (*p* < 0.05) are presented (Figure 4, Figure 5, Figure 6, Figure 7 and Figure 8).

In luminal A subtype, overexpression of *XIAP* was significantly associated with shorter OS (Figure 4).

In HER2-negative luminal B subtype, elevated expression of *CSNK2A1*, *CXCL2* and *PLAU* was significantly associated with reduced OS (Figure 5).

In HER2-positive luminal B subtype, reduced expression of *CSNK2A1*, *CXCL2* and *TAB2* was significantly associated with shorter OS (Figure 6).

In non-luminal HER2-positive subtype, reduced expression of *TAB2* and elevated expression of *PLAU* were significantly associated with shorter OS (Figure 7).

In TNBC, elevated expression of *BCL2L1*, *CSNK2A1*, *PLAU*, *XIAP*, along with reduced expression of *TAB2*, were significantly associated with shorter OS (Figure 8).

## 3. Discussion

In our study, we investigated the expression of selected NF-κB pathway components across five molecular subtypes of breast cancer: luminal A, HER2-negative luminal B, HER2-positive luminal B, non-luminal HER2-positive, TNBC. A consistent upregulation for *BCL2L1*, *CSNK2A1*, *CXCL2*, *MAP3K7*, *PLAU*, *TAB2*, *TNFAIP3*, and *XIAP* was observed at both mRNA and protein levels. These alterations were independent of molecular subtype, suggesting that key components of the NF-κB signaling pathway play an important role in breast cancer biology. Further analysis revealed several miRNAs potentially regulating these genes. Importantly, the identified miRNA–mRNA pairs consistently demonstrated inverse expression patterns, with downregulated miRNAs corresponding to upregulated target genes. This relationship is consistent with canonical miRNA-mediated repression and suggests a loss of post-transcriptional regulatory control.

*MAP3K7*, also known as transforming growth factor (TGF)-β-activated kinase 1 (*TAK1*), is a multifunctional kinase that can respond to a wide range of stimuli. It plays a central role in transducing signals from pro-inflammatory mediators such as tumor necrosis factor (TNF), interleukin (IL), and Toll-like receptor (TLR) ligands to effectors of the canonical NF-κB pathway [21]. As a result, various biological responses are triggered, including cell survival, proliferation, differentiation, innate and adaptive immunity [22]. Moreover, it has been linked to both tumor promotion and suppression depending on cell type and specific receptors [23,24]. Overexpression of *MAP3K7* has been documented in multiple cancers including ovarian [25], thyroid [26], esophageal [27], gastric cancer [28]. In breast cancer, Huang et al. reported frequent and high expression of *MAP3K7*, implicating its involvement in promoting tumor growth, metastasis, and treatment resistance [29]. Similarly, Sun et al. demonstrated its role in TNBC progression and metastasis, with nanoparticle-mediated delivery of LGALS3BP effectively inhibiting *MAP3K7* activity, suppressing tumor growth and lung metastasis [30]. Our findings align with these studies, indicating that *MAP3K7* overexpression may represent a key mechanism of NF-κB pathway activation in breast cancer. Our miRNA profiling identified miR-1297 and miR-30a as potential post-transcriptional regulators of *MAP3K7*, both significantly downregulated across all subtypes. miR-1297 has been described as tumor-suppressive in breast cancer, particularly in TNBC [31]. In addition, Mosapour et al. reported that low miR-1297 levels are associated with reduced *VLDLR* expression in malignant breast tumors and promoted tumorigenesis [32]. Contrary to these and our findings, Li et al. suggested that downregulation of miR-1297 inhibited EMT and proliferation while inducing apoptosis in the MDA-MB-231 breast cancer cell line [33]. miR-30a has been shown to inhibit EMT and improve the sensitivity of MDA-MB-231 cells to docetaxel treatment [34]. Additional studies reported that miR-30a directly targets *NOTCH1*, *SNAI1*, and *SOX4*, reducing proliferation, invasion, and metastasis [35,36,37]. Together, our findings point to a consistent pattern of *MAP3K7* overexpression, may be associated with reduced miR-1297 and miR-30a levels, suggesting a potential loss of post-transcriptional regulation, which may contribute to increased expression of NF-κB-related components and drives subtype-independent oncogenic processes in breast cancer.

*TAB2* is a key adaptor protein that links TNF receptor associated factor 6 (TRAF6) and *MAP3K7*, facilitating activation of the *MAP3K7* complex in response to TNF-α, IL-1, and TLR signaling [38]. It also acts as a mediator of drug resistance and may be a potential target for reversing tamoxifen resistance and enhancing antiestrogenic activity in breast cancer [39,40]. In our study, *TAB2* upregulation was accompanied by downregulation of miR-134 across all cancer subtypes. The observed low levels of this miRNA are consistent with previous studies in breast cancer. miR-134 has been described as tumor-suppressive, reducing proliferation, migration, and invasion, and increasing sensitivity to chemotherapy [41,42,43]. This consistent miRNA–mRNA expression pattern may reflect a regulatory mechanism facilitating aberrant NF-κB activation in breast cancer. This inverse miRNA–mRNA relationship supports a model of derepression, in which reduced miR-134 levels may contribute to increased *TAB2* expression and altered signaling dynamics within the NF-κB pathway.

*TNFAIP3*, encoding the ubiquitin-editing enzyme A20, was consistently overexpressed across all breast cancer subtypes. A20 limits inflammatory signaling through its deubiquitinating activity, acting as a brake on NF-κB activation [44]. In certain oncogenic settings, including breast cancer, A20 has been reported to promote tumor cell survival, modulate apoptotic thresholds, and contribute to therapy resistance despite its canonical inhibitory function within NF-κB signaling [45,46,47]. This apparent paradox may be explained by several mechanisms. First, A20 can selectively regulate specific branches of NF-κB signaling rather than globally suppressing pathway activity [48,49]. Second, its antiapoptotic functions—independent of NF-κB inhibition—may provide a survival advantage to tumor cells under stress conditions [50]. Third, dysregulated upstream signaling or constitutive pathway activation may override A20-mediated feedback inhibition, resulting in simultaneous overexpression of both NF-κB components and its regulatory inhibitors [51,52].

In our study, *TNFAIP3* was consistently overexpressed across all breast cancer subtypes, accompanied by downregulation of miR-125b, a potential upstream regulator. This inverse expression pattern suggests a possible loss of miRNA-mediated control contributing to elevated *TNFAIP3* levels. Importantly, given the lack of direct functional assays assessing NF-κB activity, our findings do not allow us to determine whether *TNFAIP3* overexpression reflects compensatory feedback, altered signaling dynamics, or a pro-survival role independent of canonical NF-κB inhibition. Therefore, *TNFAIP3* should be interpreted as a context-dependent regulator within a complex signaling network rather than a strictly inhibitory component in this experimental setting. Previous studies indicate that miR-125b levels are typically downregulated in breast cancer, particularly in ER-positive and metastatic tumors. Although its expression was low in most cell lines (e.g., MCF-7, T47D), it may be upregulated in highly metastatic cells, such as MDA-MB-231, suggesting a complex, context-dependent role as both a tumor suppressor and promoter [53]. miR-125b acts by inhibiting the translation of target genes, including *STARD13*, *MUC1*, *ENPEP*, *CSNK2A1*, *CCNJ*, and *MEGF9*, whose overexpression correlated with low levels of miR-125b [54,55,56]. In TNBC, it inhibited EMT by targeting *MAP2K7*, the expression of which increased with low levels of this miRNA [57].

Our study also revealed a significant overexpression of *CSNK2A1* in all breast cancer subtypes. This serine/threonine kinase phosphorylates components of several pro-survival pathways and regulates cell cycle progression, DNA repair, and resistance to apoptosis [58]. In breast cancer, increased expression of *CSNK2A1* has been associated with more aggressive disease and shorter overall and relapse-free survival. Its knockdown or inhibition reduced proliferation, invasiveness, and migratory capacity of breast cancer cells in vitro [59,60]. Importantly, pharmacological *CSNK2A1* inhibition (e.g., CX-4945/silmitasertib) or genetic silencing suppressed NF-κB activity and its target gene transcription in proliferating breast cancer cells [60]. It is worth mentioning that downregulation of *CSNK2A1* has been shown to induce NF-κB activation in the context of cell senescence. The specific context of cell growth (proliferation vs. senescence) may influence the mechanism by which *CSNK2A1* regulates NF-κB activity [61]. Furthermore, *CSNK2A1* inhibition attenuated various pro-survival signaling pathways, including NF-κB, PI3K/Akt/mTOR, and JAK/STS. Given that *CSNK2A1* underlies multidrug resistance and its inhibition leads to decreased cell viability, cell cycle arrest, and apoptosis in breast cancer cells, this suggests that *CSNK2A1* inhibition may increase sensitivity to chemotherapeutic drugs [60]. In our miRNA profiling, none of the candidate miRNAs meeting our criteria were predicted to regulate *CSNK2A1*.

In the case of *BCL2L1*, encoding the antiapoptotic protein Bcl-xL, we observed its overexpression regardless of the cancer subtype, but also none of the candidate miRNAs meeting our criteria were predicted to regulate its expression. Bcl-xL plays a key role in inhibiting mitochondrial apoptosis, thereby increasing cell survival and contributing to resistance to chemotherapeutic drugs such as taxanes and anthracyclines [62,63,64]. Elevated Bcl-xL expression increased cell viability under stress and promoted metastatic potential [62,65]. It is commonly associated with a poorer prognosis in both ER-positive breast cancer and TNBC [65,66,67]. Pharmacological blockade of Bcl-xL using BH3 mimetics (e.g., ABT-737, A-1155463) or genetic silencing induced apoptosis and sensitized breast cancer cells to chemotherapy, highlighting its therapeutic importance [62,64,66].

*XIAP* is an endogenous inhibitor of caspases-3/-7/-9, suppressing apoptosis and promoting cell survival [68,69]. Elevated *XIAP* expression in breast cancer has been associated with chemoresistance, enhanced tumor aggressiveness, and poorer disease-free survival [69,70]. These observations are consistent with our study, which demonstrated increased *XIAP* expression, peaking in TNBC, where it was additionally associated with poor OS. We further identified its potential association with miR-4329, whose expression was decreased in all breast cancer subtypes. Previous studies lack information on miR-4329 and its role in cancer, particularly breast cancer. Zhou et al. demonstrated overexpression of miR-4329 in ovarian cancer cells [71]. Higher levels of this miRNA were also noted in pseudoexfoliating glaucoma [72], whereas lower levels were observed in acute myocardial infarction [73]. Our findings suggest that reduced miR-4329 levels may contribute to increased *XIAP* expression and may influence NF-κB-related signaling dynamics, and promotes apoptosis evasion in a subtype-independent manner.

*CXCL2* is a potent neutrophil chemoattractant and key mediator of inflammatory signaling within the tumor microenvironment [74]. It engages CXCR2 receptors to activate pathways such as ERK/MAPK, PI3K/Akt, JAK/STAT3, and NF-κB, promoting tumorigenesis, invasion, immune evasion [74,75,76]. Wang et al. identified *CXCL2* as a potential biomarker and therapeutic target for breast cancer [77]. These observations are consistent with our results, as *CXCL2* was upregulated in all breast cancer subtypes included in our study. Although we did not identify any miRNAs targeting *CXCL2*, its overexpression likely reflects transcriptional induction by NF-κB or other pro-inflammatory transcription factors in the tumor stroma. Elevated *CXCL2* levels may contribute to a tumor-promoting microenvironment through sustained neutrophil and macrophage recruitment, chronic inflammation, and extracellular matrix remodeling.

In the case of *PLAU*, we reported its overexpression regardless of the cancer subtype. *PLAU* is a serine protease that converts plasminogen to plasmin, initiating extracellular matrix degradation and facilitating cell migration, invasion, and metastasis [78,79]. In breast cancer, elevated *PLAU* has been correlated with increased aggressiveness, resistance to hormone therapy, and poor clinical outcome [79,80,81]. We did not identify miRNA regulators of *PLAU* within our criteria, indicating that its increase in expression may result from transcriptional activation induced by inflammatory stimuli or other epigenetic mechanisms.

Despite the growing interest in miRNA-based therapeutic strategies, their clinical translation remains challenging [82,83]. The main limitations include the instability of miRNAs in circulation, susceptibility to rapid degradation by nucleases, and difficulties in achieving efficient and targeted delivery to tumor tissues. Various delivery systems, such as lipid nanoparticles, viral vectors, and exosome-based carriers, are currently being investigated to overcome these barriers; however, issues related to off-target effects, immune activation, and toxicity still limit their widespread clinical application [84,85,86]. Although several miRNA-based therapeutics have entered early-phase clinical trials, none have yet been broadly implemented in routine oncological practice [87,88]. Therefore, while our findings highlight potential miRNA–mRNA interactions of therapeutic relevance, further research is required to validate these targets and to develop safe and effective delivery strategies.

Several limitations of this study should be acknowledged. First, although we performed a comprehensive integrative analysis of mRNA and miRNA expression, the proposed miRNA–mRNA interactions are based on bioinformatic prediction and inverse expression patterns and therefore remain putative. Functional validation, such as luciferase reporter assays or gain- and loss-of-function experiments, will be necessary to confirm direct regulatory relationships and establish causality. Second, the study did not include direct assessment of NF-κB pathway activity, such as nuclear translocation, DNA-binding assays, or transcriptional reporter analyses. As a result, our findings do not provide mechanistic evidence of NF-κB activation, but rather reflect coordinated changes in the expression of pathway-related components. Third, although statistical approaches robust to unequal group sizes were applied, the imbalance in subtype-specific sample sizes may still influence the interpretation of subtype-level differences, and therefore these findings should be interpreted with caution.

In addition, survival analyses were conducted using external datasets rather than the primary study cohort due to the lack of long-term follow-up data, which may introduce potential confounding and limit direct clinical correlation. From a translational perspective, although our findings suggest potential relevance of miRNA–mRNA regulatory networks, the clinical application of miRNA-based therapeutics remains challenging. Limitations related to instability in circulation, inefficient and non-specific delivery, as well as potential off-target and immunogenic effects, continue to hinder their routine use in oncology. Finally, the study cohort consisted exclusively of Polish women, which may limit the generalizability of the results to more diverse populations, given potential genetic, environmental, and lifestyle-related differences influencing both NF-κB signaling and miRNA expression profiles.

## 4. Materials and Methods

### 4.1. Patients

405 patients with different subtypes of breast cancer were included in this and our previous studies [89,90,91]. 130 samples were classified as luminal A, 100 samples as HER2-negative luminal B, 96 samples as HER2-positive luminal B, 36 samples as non-luminal HER2-positive, 43 samples as triple-negative breast cancer (TNBC). During surgery, healthy tissue margin samples were collected (control group). All patients in the study were classified as T1N0M0. Patient characteristics regarding grading, age and BMI have been presented in detail in our previously published works [89,90].

The study was conducted in accordance with the 2013 Helsinki Declaration and was approved on March 10, 2023 by the Bioethical Committee of the Regional Medical Chamber in Krakow (81/KBL/OIL/2023). Informed consent was obtained from all patients.

### 4.2. Total Ribonucleic Acid (RNA) Extraction and Quality Assessment

Total RNA was isolated from tissue samples using TRIzol Reagent (Invitrogen Life Technologies, Carlsbad, CA, USA; cat. no. 15596026) following the manufacturer’s protocol. The resulting RNA was further purified using the RNeasy mini kit (QIAGEN, Hilden, Germany; cat. no. 74104) in combination with DNase I treatment to residual genomic DNA (Fermentas International Inc., Burlington, ON, Canada; cat. no. 18047019). RNA integrity was verified by 1% agarose gel electrophoresis, and RNA quantity and purity were evaluated via spectrophotometric measurement of absorbance. To ensure objective RNA quality assessment, RNA integrity was determined using the RNA Integrity Number (RIN) via capillary electrophoresis on the Agilent 2100 Bioanalyzer (Agilent Technologies, Santa Clara, CA, USA). Only samples with RIN ≥ 7.0 were used for downstream molecular analyses.

### 4.3. mRNA Microarrays

Transcriptomic profiling was conducted using the HG-U133A 2.0 microarrays (Affymetrix, Santa Clara, CA, USA) with the GeneChip™ 3′IVT PLUS kit (Thermo Fisher Scientific, Inc., Waltham, MA, USA; cat. no. 902416), according to the manufacturer’s guidelines. To define the gene panel related to NF-κB activity, the KEGG signaling pathway database was queried for pathway hsa04064 (NF-κB signaling). This yielded a set of 105 genes, represented by 260 mRNA probe sets on the selected microarray platform. The approach of KEGG-driven gene panel selection was applied in our previous studies [89,90].

### 4.4. Validation by RT-qPCR

To validate microarray findings, expression levels of eight genes exhibiting significant deregulation across all breast cancer subtypes were assessed by reverse transcription quantitative PCR (RT-qPCR). The SensiFast SYBR No-ROX One-Step Kit (Bioline, London, UK) was employed following the manufacturer’s protocol. The selected genes included BCL2 like 1 (*BCL2L1*), casein kinase 2 alpha 1 (*CSNK2A1*), C-X-C motif chemokine ligand 2 (*CXCL2*), mitogen-activated protein kinase kinase kinase 7 (*MAP3K7*), urokinase (*PLAU*), TGF-beta activated kinase 1 (*MAP3K7*) binding protein 2 (*TAB2*), tumor necrosis factor alpha-induced protein 3 (*TNFAIP3*), X-linked inhibitor of apoptosis protein (*XIAP*) (Table 4). Relative gene expression was calculated using the 2^−ΔΔCt^ method, with β-actin (*ACTB*) serving as an endogenous control.

### 4.5. Protein Quantification by ELISA

Quantitative analysis of protein expression was conducted using enzyme-linked immunosorbent assay (ELISA). Commercially available kits (MyBioSource, San Diego, CA, USA) were used to assess levels of the following proteins: *BCL2L1* kit (cat. no. MBS9392826), *CSNK2A1* kit (cat. no. MBS763078), *CXCL2* kit (cat. no. MBS2880010), *MAP3K7* kit (cat. no. MBS7612749), *PLAU* kit (cat. no. MBS721191), *TAB2* kit (cat. no. MBS762519), *TNFAIP3* kit (cat. no. MBS2881407), *XIAP* kit (cat. no. MBS161168).

### 4.6. miRNA Profiling and Target Prediction

Differentially expressed miRNAs distinguishing tumor tissue from adjacent non-tumor controls were identified using the Affymetrix miRNA Microarray 2.0 platform. Sample processing was conducted with the FlashTag Biotin HSR RNA Labeling Kit and the Hybridization, Wash, and Stain Kit (all from Affymetrix, Santa Clara, CA, USA), following the manufacturer’s instructions.

To predict miRNAs potentially regulating the expression of *BCL2L1*, *CSNK2A1*, *CXCL2*, *MAP3K7*, *PLAU*, *TAB2*, *TNFAIP3*, and *XIAP*, the miRDB tool (http://mirdb.org) was used. Only targets with a confidence score of ≥80 were retained for further analysis to enhance prediction specificity [92]. In addition, predicted miRNA–mRNA interactions were cross-validated using the TargetScan database (https://www.targetscan.org, accessed on 1 April 2025) to increase the reliability of the identified regulatory relationships [93].

### 4.7. Statistical Analysis

Transcriptomic data obtained from HG-U133A 2.0 microarrays (Affymetrix, Santa Clara, CA, USA) were analyzed using Transcriptome Analysis Console (Thermo Fisher Scientific, Waltham, MA, USA). Raw microarray data were processed using the Robust Multiarray Average (RMA) algorithm implemented in RMA Express (v1.20.0, Affymetrix), which includes background correction, quantile normalization to ensure comparable signal distribution across arrays, and summarization of probe-level data using the median polish algorithm. Expression values were log2-transformed to stabilize variance and improve comparability between samples. The analysis included fluorescence signals from all probe types (“_at”, “_s_at”, and “_x_at”), representing transcript-specific, splice-variant, and cross-hybridizing probes, respectively. Microarray analysis was performed on independent biological samples representing tumor and matched adjacent non-cancerous tissues, with samples stratified according to molecular subtype. No technical replicates were applied.

Differential expression analysis was performed using one-way analysis of variance (ANOVA), followed by Tukey’s post hoc test (*p* < 0.05). To control for multiple hypothesis testing, the Benjamini–Hochberg procedure was applied, and the False Discovery Rate (FDR) was calculated. Transcripts were considered significantly differentially expressed when they met both statistical significance criteria (adjusted *p*-value, FDR < 0.05) and fold-change thresholds (FC > 2 or FC < −2).

The fold-change threshold was selected to prioritize robust and biologically meaningful expression differences while reducing potential false-positive findings inherent to high-throughput transcriptomic analyses. Due to unequal sample sizes across breast cancer subtypes, ANOVA was selected as a method robust to moderate group size imbalance. The assumption of homogeneity of variances was verified using Levene’s test.

Statistica 13.3 (StatSoft, Krakow, Poland) was used to analyze the RT-qPCR and ELISA results. Data distribution was assessed using the Shapiro–Wilk test, which indicated non-normality and justified the use of non-parametric Kruskal–Wallis and Dunn’s multiple comparison tests.

G*Power 3.1.9.718 was used to estimate the sample size [94]. One-way ANOVA (f = 0.25, α = 0.05, power = 0.95) estimated a sample size of 305. Since the study included 405 patients, a post hoc test was performed, which showed that with this sample size, the power was 0.99.

Overall survival (OS) was analyzed across all molecular subtypes using the Kaplan–Meier plotter (http://kmplot.com/; accessed: 17 June 2025) [95,96], which integrates gene expression and survival data from multiple independent cohorts. Patients were stratified into high- and low-expression groups based on the median expression level of the indicated gene. Survival differences were assessed using the log-rank test. Hazard ratios (HR) with 95% confidence intervals (CI) are presented where available. Censored observations are indicated by tick marks. The follow-up period was limited to 60 months. The primary study cohort was not suitable for survival analysis due to the limited availability of long-term follow-up and outcome data; therefore, external validation was applied to assess the clinical relevance of the identified molecular alterations.

## 5. Conclusions

This study provides a comprehensive analysis of the expression of NF-κB-related genes and their potential miRNA regulators across five molecular subtypes of breast cancer in a Polish patient cohort. We observed consistent upregulation of eight key genes (*BCL2L1*, *CSNK2A1*, *CXCL2*, *MAP3K7*, *PLAU*, *TAB2*, *TNFAIP3*, and *XIAP*) at both the mRNA and protein levels, regardless of subtype. In parallel, several miRNAs (miR-1297, miR-30a, miR-134, miR-125b, and miR-4329) were consistently downregulated and may be involved in shaping these expression patterns through post-transcriptional mechanisms.

Taken together, these findings point to a shared molecular profile involving components of the NF-κB pathway and their potential regulatory miRNAs across breast cancer subtypes. However, it is important to emphasize that our conclusions are based on expression data and bioinformatic predictions. As we did not directly assess NF-κB activity—such as nuclear translocation, DNA binding, or transcriptional activation—our results should not be interpreted as providing direct mechanistic evidence of pathway activation.

Nevertheless, the consistent miRNA–mRNA expression patterns identified in this study offer a valuable framework for future research. Further functional studies will be essential to confirm these regulatory relationships and to better understand the role of NF-κB signaling in breast cancer progression. In this context, our findings may help guide the identification of novel molecular targets and support the development of more targeted therapeutic strategies.

## Figures and Tables

**Table 1:** Differential expression (fold change, FC) of NF-κB-related mRNAs across breast cancer subtypes compared to control tissue. Only probes meeting statistical significance criteria (adjusted *p* < 0.05; |FC| > 2) are presented.

ID	mRNA	Fold Change				
		LumA vs. C	HER2-Negative LumB vs. C	HER2-Positive LumB vs. C	Non-Luminal HER2-Positive vs. C	TNBC vs. C
206665_s_at	*BCL2L1*	3.1	3.21	3.03	3.1	5.40
215037_s_at	*BCL2L1*	3.17	2.90	2.87	3.14	3.31
206075_s_at	*CSNK2A1*	3.51	4.02	3.68	5.05	5.10
212073_at	*CSNK2A1*	2.26	2.76	2.38	3.45	2.66
212075_s_at	*CSNK2A1*	2.12	2.30	2.37	3.03	2.81
1567014_s_at	*CSNK2A1*	3.17	3.23	2.90	4.67	2.38
209774_x_at	*CXCL2*	2.46	2.69	2.72	3.28	3.14
211536_x_at	*MAP3K7*	2.70	2.26	2.48	3.78	6.05
211537_x_at	*MAP3K7*	2.69	2.17	2.44	3.77	2.06
206854_s_at	*MAP3K7*	2.01	2.01	2.03	2.54	2.76
205479_s_at	*PLAU*	2.62	3.18	3.33	3.97	2.35
211668_s_at	*PLAU*	4.59	4.54	7.09	7.76	8.53
210284_s_at	*TAB2*	4.16	3.59	4.13	4.97	4.89
202643_s_at	*TNFAIP3*	2.87	2.36	2.37	4.13	4.97
225859_at	*XIAP*	3.10	3.22	2.85	2.91	4.06
243026_x_at	*XIAP*	2.76	2.60	2.55	3.01	7.48

Data are presented as fold change (FC) relative to control tissue. Statistical significance was determined using one-way ANOVA followed by Tukey’s post hoc test, with false discovery rate (FDR) correction (Benjamini–Hochberg). Adjusted *p*-values < 0.05 were considered significant. ID, number of the probe; LumA, luminal A; LumB, luminal B; HER2, human epidermal growth factor receptor 2; TNBC, triple-negative breast cancer; C, control; *BCL2L1*, BCL2 like 1; *CSNK2A1*, casein kinase 2 alpha 1; *CXCL2*, C-X-C motif chemokine ligand 2; *MAP3K7*, mitogen-activated protein kinase kinase kinase 7; *PLAU*, urokinase; *TAB2*, TGF-beta activated kinase 1 (*MAP3K7*) binding protein 2; *TNFAIP3*, tumor necrosis factor alpha-induced protein 3; *XIAP*, X-linked inhibitor of apoptosis protein.

**Table 2:** Concentration of *BCL2L1*, *CSNK2A1*, *CXCL2*, *MAP3K7*, *PLAU*, *TAB2*, *TNFAIP3*, *XIAP* in breast cancer subtypes and control group (*p* < 0.05).

Protein [ng/mL]	Control	LumA	HER2-Negative LumB	HER2-Positive LumB	HER2-Positive	TNBC
*BCL2L1*	9.57 ± 0.31	17.46 ± 0.19 *	19 ± 0.27 *	19.91 ± 0.27 *	19.51 ± 0.34 *	24.46 ± 0.31 *
*CSNK2A1*	1.18 ± 0.19	3.08 ± 0.17 *	4.46 ± 0.26 *	4.39 ± 0.21 *	6.05 ± 0.34 *	6.23 ± 0.23 *
*CXCL2*	1.93 ± 0.19	3.83 ± 0.14 *	5.18 ± 0.25 *	5.19 ± 0.19 *	6.82 ± 0.34 *	7 ± 0.23 *
*MAP3K7*	1.74 ± 0.15	3.18 ± 0.2 *	3.53 ± 0.18 *	3.69 ± 0.19 *	4.25 ± 0.25 *	4.61 ± 0.16 *
*PLAU*	6.23 ± 0.13	10.93 ± 0.22 *	12.01 ± 0.19 *	12.33 ± 0.24 *	16.2 ± 0.24 *	25.19 ± 0.19 *
*TAB2*	0.09 ± 0.01	0.22 ± 0.01 *	0.3 ± 0.01 *	0.31 ± 0.01 *	0.44 ± 0.01 *	0.48 ± 0.01 *
*TNFAIP3*	5.2 ± 0.18	12.12 ± 0.13 *	12.29 ± 0.16 *	12.3 ± 0.19 *	17.78 ± 0.15 *	25.6 ± 0.21 *
*XIAP*	2.92 ± 0.08	4.98 ± 0.09 *	4.99 ± 0.17 *	5.05 ± 0.18 *	7.85 ± 0.19 *	11.68 ± 0.17 *

LumA, luminal A; LumB, luminal B; HER2, human epidermal growth factor receptor 2; TNBC, triple-negative breast cancer; C, control; *BCL2L1*, BCL2 like 1; *CSNK2A1*, casein kinase 2 alpha 1; *CXCL2*, C-X-C motif chemokine ligand 2; *MAP3K7*, mitogen-activated protein kinase kinase kinase 7; *PLAU*, urokinase; *TAB2*, TGF-beta activated kinase 1 (*MAP3K7*) binding protein 2; *TNFAIP3*, tumor necrosis factor alpha-induced protein 3; *XIAP*, X-linked inhibitor of apoptosis protein. * *p* < 0.05 vs. control.

**Table 3:** Differential expression of miRNAs potentially regulating NF-κB-related genes across breast cancer subtypes. Only miRNAs meeting significance criteria (adjusted *p* < 0.05; |FC| > 2) are shown.

mRNA	miRNA	Target Score	Fold Change				
			LumA vs. C	HER2-Negative LumB vs. C	HER2-Positive LumB vs. C	HER2-Positive vs. C	TNBC vs. C
*MAP3K7*	miR-1297	90	−2.10 *	−3.21 *	−3.45 *	−4.97 *	−5.62 *
	miR-30a	83	−2.14 *	−2.64 *	−2.90 *	−4.39 *	−5.84 *
*TAB2*	miR-134	95	−4.9 *	−5.75 *	−6.52 *	−7.09 *	−8.44 *
*TNFAIP3*	miR-125b	84	−2.03 *	−2.22 *	−2.6 *	−3.61 *	−3.65 *
*XIAP*	miR-4329	94	−2.13 *	−2.63 *	−3.01 *	−4.16 *	−4.97 *

Data are presented as fold change (FC) relative to control tissue. Negative values indicate downregulation. Statistical significance was determined using one-way ANOVA with FDR correction (Benjamini–Hochberg). Adjusted *p*-values < 0.05 were considered significant. Target scores were obtained from the miRDB database. LumA, luminal A; LumB, luminal B; HER2, human epidermal growth factor receptor 2; TNBC, triple-negative breast cancer; C, control; *BCL2L1*, BCL2 like 1; *CSNK2A1*, casein kinase 2 alpha 1; *CXCL2*, C-X-C motif chemokine ligand 2; *MAP3K7*, mitogen-activated protein kinase kinase kinase 7; *PLAU*, urokinase; *TAB2*, TGF-beta activated kinase 1 (*MAP3K7*) binding protein 2; *TNFAIP3*, tumor necrosis factor alpha-induced protein 3; *XIAP*, X-linked inhibitor of apoptosis protein. * *p* < 0.05 vs. control.

**Table 4:** RT-qPCR primers.

mRNA	RT-qPCR Primers (5′-3′)
*BCL2L1*	Forward: GCCACTTACCTGAATGACCACC Reverse: AACCAGCGGTTGAAGCGTTCCT
*CSNK2A1*	Forward: GGTGAGGATAGCCAAGGTTCTG Reverse: TCACTGTGGACAAAGCGTTCCC
*CXCL2*	Forward: GGCAGAAAGCTTGTCTCAACCC Reverse: CTCCTTCAGGAACAGCCACCAA
*MAP3K7*	Forward: CAGAGCAACTCTGCCACCAGTA Reverse: CATTTGTGGCAGGAACTTGCTCC
*PLAU*	Forward: GGCTTAACTCCAACACGCAAGG Reverse: CCTCCTTGGAACGGATCTTCAG
*TAB2*	Forward: TATTCAGCACCTCACGGACCCT Reverse: CTTTGAAGTCGTTCCATTCTGGC
*TNFAIP3*	Forward: CTCAACTGGTGTCGAGAAGTCC Reverse: TTCCTTGAGCGTGCTGAACAGC
*XIAP*	Forward: TGGCAGATTATGAAGCACGGATC Reverse: AGTTAGCCCTCCTCCACAGTGA
*ACTB*	Forward: TCACCCACACTGTGCCCATCTACGA Reverse: CAGCGGAACCGCTCATTGCCAATGG

*BCL2L1*, BCL2 like 1; *CSNK2A1*, casein kinase 2 alpha 1; *CXCL2*, C-X-C motif chemokine ligand 2; *MAP3K7*, mitogen-activated protein kinase kinase kinase 7; *PLAU*, urokinase; *TAB2*, TGF-beta activated kinase 1 (*MAP3K7*) binding protein 2; *TNFAIP3*, tumor necrosis factor alpha-induced protein 3; *XIAP*, X-linked inhibitor of apoptosis protein; *ACTB*, β-actin.

## Data Availability

The data used to support findings of this study are included in this article.

## Abbreviations

The following abbreviations are used in this manuscript:

ACTB	Beta-actin
ANOVA	Analysis of variance
*BCL2L1*	BCL2 like 1
BMI	Body mass index
C	Control
*CSNK2A1*	Casein kinase 2 alpha 1
*CXCL2*	C-X-C motif chemokine ligand 2
DNA	Deoxyribonucleic acid
ELISA	Enzyme-linked immunosorbent assay
EMT	Epithelial–mesenchymal transition
ER	Estrogen receptor
FC	Fold change
HER2	Human epidermal growth factor receptor 2
IκBα	Inhibitor of kappa B alpha
IKK	IκB kinase
IL	Interleukin
KEGG	Kyoto Encyclopedia of Genes and Genomes
*MAP3K7*	Mitogen-activated protein kinase kinase kinase 7
miRNA	MicroRNA
mRNA	Messenger ribonucleic acid
NF-κB	Nuclear factor kappa B
NIK	NF-κB-inducing kinase
OS	Overall survival
*PLAU*	Plasminogen activator, urokinase
PR	Progesterone receptor
qPCR	Quantitative polymerase chain reaction
RELA	v-rel avian reticuloendotheliosis viral oncogene homolog A
RNA	Ribonucleic acid
RT-qPCR	Reverse transcription quantitative polymerase chain reaction
*TAB2*	TGF-beta activated kinase 1 (*MAP3K7*) binding protein 2
TAK1	TGF-beta activated kinase 1
TGF-β	Transforming growth factor beta
TLR	Toll-like receptor
TNBC	Triple-negative breast cancer
TNF	Tumor necrosis factor
*TNFAIP3*	Tumor necrosis factor alpha-induced protein 3
TRAF6	TNF receptor-associated factor 6
VLDLR	Very low-density lipoprotein receptor
*XIAP*	X-linked inhibitor of apoptosis protein

## References

[1] Bray F., Laversanne M., Sung H., Ferlay J., Siegel R.L., Soerjomataram I., Jemal A. (2024). Global Cancer Statistics 2022: GLOBOCAN Estimates of Incidence and Mortality Worldwide for 36 Cancers in 185 Countries. CA Cancer J. Clin..

[2] https://onkologia.org.pl/sites/default/files/publications/2024-01/0_krn-2023-book-2024-01-22.pdf.

[3] Yang X., Smirnov A., Buonomo O.C., Mauriello A., Shi Y., Bischof J., Woodsmith J., TOR CENTRE, Melino G., Candi E. (2023). A Primary Luminal/HER2 Negative Breast Cancer Patient with Mismatch Repair Deficiency. Cell Death Discov..

[4] Łukasiewicz S., Czeczelewski M., Forma A., Baj J., Sitarz R., Stanisławek A. (2021). Breast Cancer-Epidemiology, Risk Factors, Classification, Prognostic Markers, and Current Treatment Strategies—An Updated Review. Cancers.

[5] Thomas A., Reis-Filho J.S., Geyer C.E., Wen H.Y. (2023). Rare Subtypes of Triple Negative Breast Cancer: Current Understanding and Future Directions. npj Breast Cancer.

[6] Zhang T., Ma C., Zhang Z., Zhang H., Hu H. (2021). NF-κB Signaling in Inflammation and Cancer. MedComm.

[7] Guo Q., Jin Y., Chen X., Ye X., Shen X., Lin M., Zeng C., Zhou T., Zhang J. (2024). NF-κB in Biology and Targeted Therapy: New Insights and Translational Implications. Signal Transduct. Target. Ther..

[8] Mao H., Zhao X., Sun S. (2025). NF-κB in Inflammation and Cancer. Cell. Mol. Immunol..

[9] Oh A., Pardo M., Rodriguez A., Yu C., Nguyen L., Liang O., Chorzalska A., Dubielecka P.M. (2023). NF-κB Signaling in Neoplastic Transition from Epithelial to Mesenchymal Phenotype. Cell Commun. Signal..

[10] Wu X., Sun L., Xu F. (2023). NF-κB in Cell Deaths, Therapeutic Resistance and Nanotherapy of Tumors: Recent Advances. Pharmaceuticals.

[11] Guo Q., Jin Y., Lin M., Zeng C., Zhang J. (2024). NF-κB Signaling in Therapy Resistance of Breast Cancer: Mechanisms, Approaches, and Challenges. Life Sci..

[12] Cao Y., Yi Y., Han C., Shi B. (2024). NF-κB Signaling Pathway in Tumor Microenvironment. Front. Immunol..

[13] Wibisana J.N., Okada M. (2022). Encoding and Decoding NF-κB Nuclear Dynamics. Curr. Opin. Cell Biol..

[14] Bacher S., Meier-Soelch J., Kracht M., Schmitz M.L. (2021). Regulation of Transcription Factor NF-κB in Its Natural Habitat: The Nucleus. Cells.

[15] Prezioso C., Limongi D., Checconi P., Ciotti M., Legramante J.M., Petrangeli C.M., Leonardis F., Giovannelli A., Terrinoni A., Bernardini S. (2024). Role of miR-9 in Modulating NF-κB Signaling and Cytokine Expression in COVID-19 Patients. Int. J. Mol. Sci..

[16] Liu M., Wang D., Yan Z., Zhou M. (2025). MiR-298 Suppresses Astrocytic NF-κB Activity and Neuroinflammation via Targeting MyD88 in Bone Cancer Pain. Korean J. Pain.

[17] Wang G., Yang Q., Han Y., Zhang Y., Pan W., Ma Z., Tian H., Qu X. (2025). miR-32-5p Suppresses the Progression of Hepatocellular Carcinoma by Regulating the GSK3β/NF-κB Signaling. Acta Biochim. Biophys. Sin..

[18] Chen Y., Gao B., Pan Y., Wang Q., Zhang Q. (2024). MiR-525-5p Modulates Cell Proliferation, Cell Cycle, and Apoptosis in Burkitt’s Lymphoma by Targeting MyD88 and Regulating the NF-κB Signaling Pathway. Ann. Hematol..

[19] Liu S., He Q., Zhang N., Li Y., Liu Q. (2025). MicroRNA-3135b as a Therapeutic Target and Clinical Biomarker for Stroke: Regulation of the NF-κB/IKKβ Signaling Pathway. Mol. Neurobiol..

[20] Chen R., Yang M., Huang W., Wang B. (2021). Cascades between miRNAs, lncRNAs and the NF-κB Signaling Pathway in Gastric Cancer (Review). Exp. Ther. Med..

[21] Benedik N.S., Proj M., Steinebach C., Sova M., Sosič I. (2025). Targeting TAK1: Evolution of Inhibitors, Challenges, and Future Directions. Pharmacol. Ther..

[22] Iacobazzi D., Convertini P., Todisco S., Santarsiero A., Iacobazzi V., Infantino V. (2023). New Insights into NF-κB Signaling in Innate Immunity: Focus on Immunometabolic Crosstalks. Biology.

[23] Sheng N., Shindo K., Ohuchida K., Shinkawa T., Zhang B., Feng H., Yamamoto T., Moriyama T., Ikenaga N., Nakata K. (2024). TAK1 Promotes an Immunosuppressive Tumor Microenvironment through Cancer-Associated Fibroblast Phenotypic Conversion in Pancreatic Ductal Adenocarcinoma. Clin. Cancer Res..

[24] Xu G., Niu L., Wang Y., Yang G., Zhu X., Yao Y., Zhao G., Wang S., Li H. (2022). HDAC6-Dependent Deacetylation of TAK1 Enhances sIL-6R Release to Promote Macrophage M2 Polarization in Colon Cancer. Cell Death Dis..

[25] Cai P.C.H., Shi L., Liu V.W.S., Tang H.W.M., Liu I.J., Leung T.H.Y., Chan K.K.L., Yam J.W.P., Yao K.-M., Ngan H.Y.S. (2014). Elevated TAK1 Augments Tumor Growth and Metastatic Capacities of Ovarian Cancer Cells through Activation of NF-κB Signaling. Oncotarget.

[26] Lin P., Niu W., Peng C., Zhang Z., Niu J. (2015). The Role of TAK1 Expression in Thyroid Cancer. Int. J. Clin. Exp. Pathol..

[27] Wen J., Hu Y., Luo K.-J., Yang H., Zhang S.-S., Fu J.-H. (2013). Positive Transforming Growth Factor-β Activated Kinase-1 Expression Has an Unfavorable Impact on Survival in T3N1-3M0 Esophageal Squamous Cell Carcinomas. Ann. Thorac. Surg..

[28] Yang Y., Qiu Y., Tang M., Wu Z., Hu W., Chen C. (2017). Expression and Function of Transforming Growth Factor-β-Activated Protein Kinase 1 in Gastric Cancer. Mol. Med. Rep..

[29] Huang H.-L., Chiang C.-H., Hung W.-C., Hou M.-F. (2014). Targeting of TGF-β-Activated Protein Kinase 1 Inhibits Chemokine (C-C Motif) Receptor 7 Expression, Tumor Growth and Metastasis in Breast Cancer. Oncotarget.

[30] Sun E.-G., Vijayan V., Park M.-R., Yoo K.H., Cho S.-H., Bae W.-K., Shim H.-J., Hwang J.-E., Park I.-K., Chung I.-J. (2023). Suppression of Triple-Negative Breast Cancer Aggressiveness by LGALS3BP via Inhibition of the TNF-α–TAK1–MMP9 Axis. Cell Death Discov..

[31] Hu J., Ji C., Hua K., Wang X., Deng X., Li J., Graham D., Fang L. (2020). Hsa_circ_0091074 Regulates TAZ Expression via microRNA-1297 in Triple Negative Breast Cancer Cells. Int. J. Oncol..

[32] Mosapour A., Karami Tehrani F.S., Atri M. (2020). Differential Expression of miR-1297, miR-3191-5p, miR-4435, and miR-4465 in Malignant and Benign Breast Tumors. Iran. J. Basic Med. Sci..

[33] Li H., Lian B., Liu Y., Chai D., Li J. (2020). MicroRNA-1297 Downregulation Inhibits Breast Cancer Cell Epithelial-Mesenchymal Transition and Proliferation in a FA2H-Dependent Manner. Oncol. Lett..

[34] Cheng C.-W., Liu Y.-F., Liao W.-L., Chen P.-M., Hung Y.-T., Lee H.-J., Cheng Y.-C., Wu P.-E., Lu Y.-S., Shen C.-Y. (2024). miR-622 Increases miR-30a Expression through Inhibition of Hypoxia-Inducible Factor 1α to Improve Metastasis and Chemoresistance in Human Invasive Breast Cancer Cells. Cancers.

[35] Zhang H.-D., Jiang L.-H., Sun D.-W., Li J., Tang J.-H. (2017). miR-30a Inhibits the Biological Function of Breast Cancer Cells by Targeting Notch1. Int. J. Mol. Med..

[36] Xiao B., Shi X., Bai J. (2019). miR-30a Regulates the Proliferation and Invasion of Breast Cancer Cells by Targeting Snail. Oncol. Lett..

[37] Liu Z., Mi M., Zheng X., Zhang C., Zhu F., Liu T., Wu G., Zhang L. (2021). miR-30a/SOX4 Double Negative Feedback Loop Is Modulated by Disulfiram and Regulates EMT and Stem Cell-like Properties in Breast Cancer. J. Cancer.

[38] Salauddin M., Bhattacharyya D., Samanta I., Saha S., Xue M., Hossain M.G., Zheng C. (2025). Role of TLRs as Signaling Cascades to Combat Infectious Diseases: A Review. Cell. Mol. Life Sci..

[39] Cutrupi S., Reineri S., Panetto A., Grosso E., Caizzi L., Ricci L., Friard O., Agati S., Scatolini M., Chiorino G. (2012). Targeting of the Adaptor Protein Tab2 as a Novel Approach to Revert Tamoxifen Resistance in Breast Cancer Cells. Oncogene.

[40] Brand J.S., Li J., Humphreys K., Karlsson R., Eriksson M., Ivansson E., Hall P., Czene K. (2015). Identification of Two Novel Mammographic Density Loci at 6Q25.1. Breast Cancer Res..

[41] Zhang J., Ma Y., Wang S., Chen F., Gu Y. (2015). C/EBPα Inhibits Proliferation of Breast Cancer Cells via a Novel Pathway of miR-134/CREB. Int. J. Clin. Exp. Pathol..

[42] O’Brien K., Lowry M.C., Corcoran C., Martinez V.G., Daly M., Rani S., Gallagher W.M., Radomski M.W., MacLeod R.A.F., O’Driscoll L. (2015). miR-134 in Extracellular Vesicles Reduces Triple-Negative Breast Cancer Aggression and Increases Drug Sensitivity. Oncotarget.

[43] Su X., Zhang L., Li H., Cheng P., Zhu Y., Liu Z., Zhao Y., Xu H., Li D., Gao H. (2017). MicroRNA-134 Targets KRAS to Suppress Breast Cancer Cell Proliferation, Migration and Invasion. Oncol. Lett..

[44] Sharif-Askari F.S., Al-Khayyal N., Talaat I., Sharif-Askari N.S., Rawat S., Jundi M., SyrjÄnen K., Hamoudi R., Bendardaf R. (2021). Immunohistochemical Assessment of TNFAIP3/A20 Expression Correlates with Early Tumorigenesis in Breast Cancer. Anticancer Res..

[45] Vendrell J.A., Ghayad S., Ben-Larbi S., Dumontet C., Mechti N., Cohen P.A. (2007). A20/TNFAIP3, a New Estrogen-Regulated Gene That Confers Tamoxifen Resistance in Breast Cancer Cells. Oncogene.

[46] Lee E., Ouzounova M., Piranlioglu R., Ma M.T., Guzel M., Marasco D., Chadli A., Gestwicki J.E., Cowell J.K., Wicha M.S. (2019). The Pleiotropic Effects of TNFα in Breast Cancer Subtypes Is Regulated by TNFAIP3/A20. Oncogene.

[47] Song C., Kendi A.T., Lowe V.J., Lee S. (2022). The A20/TNFAIP3-CDC20-CASP1 Axis Promotes Inflammation-Mediated Metastatic Disease in Triple-Negative Breast Cancer. Anticancer Res..

[48] Jeon H., Lee C. (2025). The Dual Role of A20 (TNFAIP3) in Viral Infection: A Context-Dependent Regulator of Immunity and Pathogenesis. Viruses.

[49] Shembade N., Harhaj E.W. (2012). Regulation of NF-κB Signaling by the A20 Deubiquitinase. Cell. Mol. Immunol..

[50] Yu H., Lin L., Zhang Z., Zhang H., Hu H. (2020). Targeting NF-κB Pathway for the Therapy of Diseases: Mechanism and Clinical Study. Signal Transduct. Target. Ther..

[51] Courtois G., Fauvarque M.-O. (2018). The Many Roles of Ubiquitin in NF-κB Signaling. Biomedicines.

[52] Prescott J.A., Mitchell J.P., Cook S.J. (2021). Inhibitory Feedback Control of NF-κB Signalling in Health and Disease. Biochem. J..

[53] Tang F., Zhang R., He Y., Zou M., Guo L., Xi T. (2012). MicroRNA-125b Induces Metastasis by Targeting STARD13 in MCF-7 and MDA-MB-231 Breast Cancer Cells. PLoS ONE.

[54] Zhang Y., Yan L.-X., Wu Q.-N., Du Z.-M., Chen J., Liao D.-Z., Huang M.-Y., Hou J.-H., Wu Q.-L., Zeng M.-S. (2011). miR-125b Is Methylated and Functions as a Tumor Suppressor by Regulating the ETS1 Proto-Oncogene in Human Invasive Breast Cancer. Cancer Res..

[55] Feliciano A., Castellvi J., Artero-Castro A., Leal J.A., Romagosa C., Hernández-Losa J., Peg V., Fabra A., Vidal F., Kondoh H. (2013). miR-125b Acts as a Tumor Suppressor in Breast Tumorigenesis via Its Novel Direct Targets ENPEP, CK2-α, CCNJ, and MEGF9. PLoS ONE.

[56] Rajabi H., Jin C., Ahmad R., McClary A.C., Joshi M.D., Kufe D. (2010). Mucin 1 Oncoprotein Expression Is Suppressed by the miR-125b Oncomir. Genes Cancer.

[57] Hong L., Pan F., Jiang H., Zhang L., Liu Y., Cai C., Hua C., Luo X., Sun J., Chen Z. (2016). miR-125b Inhibited Epithelial–Mesenchymal Transition of Triple-Negative Breast Cancer by Targeting MAP2K7. Onco Targets Ther..

[58] Halloran D., Pandit V., Nohe A. (2022). The Role of Protein Kinase CK2 in Development and Disease Progression: A Critical Review. J. Dev. Biol..

[59] Bae J.S., Park S.-H., Jamiyandorj U., Kim K.M., Noh S.J., Kim J.R., Park H.J., Kwon K.S., Jung S.H., Park H.S. (2016). CK2α/CSNK2A1 Phosphorylates SIRT6 and Is Involved in the Progression of Breast Carcinoma and Predicts Shorter Survival of Diagnosed Patients. Am. J. Pathol..

[60] Gray G.K., McFarland B.C., Rowse A.L., Gibson S.A., Benveniste E.N. (2014). Therapeutic CK2 Inhibition Attenuates Diverse Prosurvival Signaling Cascades and Decreases Cell Viability in Human Breast Cancer Cells. Oncotarget.

[61] Song J., Bae Y.-S. (2021). CK2 Down-Regulation Increases the Expression of Senescence-Associated Secretory Phenotype Factors through NF-κB Activation. Int. J. Mol. Sci..

[62] Keitel U., Scheel A., Thomale J., Halpape R., Kaulfuß S., Scheel C., Dobbelstein M. (2014). Bcl-xL Mediates Therapeutic Resistance of a Mesenchymal Breast Cancer Cell Subpopulation. Oncotarget.

[63] Vogler M., Braun Y., Smith V.M., Westhoff M.-A., Pereira R.S., Pieper N.M., Anders M., Callens M., Vervliet T., Abbas M. (2025). The BCL2 Family: From Apoptosis Mechanisms to New Advances in Targeted Therapy. Signal Transduct. Target. Ther..

[64] Yin L., Hu X., Pei G., Tang M., Zhou Y., Zhang H., Huang M., Li S., Zhang J., Citu C. (2024). Genome-Wide CRISPR Screen Reveals the Synthetic Lethality between BCL2L1 Inhibition and Radiotherapy. Life Sci. Alliance.

[65] Kawiak A., Kostecka A. (2022). Regulation of Bcl-2 Family Proteins in Estrogen Receptor-Positive Breast Cancer and Their Implications in Endocrine Therapy. Cancers.

[66] Nocquet L., Roul J., Lefebvre C.C., Duarte L., Campone M., Juin P.P., Souazé F. (2024). Low BCL-xL Expression in Triple-Negative Breast Cancer Cells Favors Chemotherapy Efficacy, and This Effect Is Limited by Cancer-Associated Fibroblasts. Sci. Rep..

[67] Alcon C., Zañudo J.G.T., Albert R., Wagle N., Scaltriti M., Letai A., Samitier J., Montero J. (2021). ER+ Breast Cancer Strongly Depends on MCL-1 and BCL-xL Anti-Apoptotic Proteins. Cells.

[68] Tu H., Costa M. (2020). XIAP’s Profile in Human Cancer. Biomolecules.

[69] Devi G.R., Finetti P., Morse M.A., Lee S., de Nonneville A., Van Laere S., Troy J., Geradts J., McCall S., Bertucci F. (2021). Expression of X-Linked Inhibitor of Apoptosis Protein (XIAP) in Breast Cancer Is Associated with Shorter Survival and Resistance to Chemotherapy. Cancers.

[70] Hussain A.R., Siraj A.K., Ahmed M., Bu R., Pratheeshkumar P., Alrashed A.M., Qadri Z., Ajarim D., Al-Dayel F., Beg S. (2017). XIAP Over-Expression Is an Independent Poor Prognostic Marker in Middle Eastern Breast Cancer and Can Be Targeted to Induce Efficient Apoptosis. BMC Cancer.

[71] Zhou Y., Zheng X., Lu J., Chen W., Li X., Zhao L. (2018). Ginsenoside 20(S)-Rg3 Inhibits the Warburg Effect Via Modulating DNMT3A/MiR-532-3p/HK2 Pathway in Ovarian Cancer Cells. Cell. Physiol. Biochem..

[72] Czop M., Gasińska K., Kosior-Jarecka E., Wróbel-Dudzińska D., Kocki J., Żarnowski T. (2023). Twenty Novel MicroRNAs in the Aqueous Humor of Pseudoexfoliation Glaucoma Patients. Cells.

[73] Chen S., Fang H., Liu R., Fang Y., Wu Z., Xie P. (2021). miR-6718-5p and miR-4329 Can Be Used as Potential Biomarkers for Acute Myocardial Infarction. J. Card. Surg..

[74] Lv Y., Chen C., Han M., Tian C., Song F., Feng S., Xu M., Zhao Z., Zhou H., Su W. (2025). CXCL2: A Key Player in the Tumor Microenvironment and Inflammatory Diseases. Cancer Cell Int..

[75] Xu H., Lin F., Wang Z., Yang L., Meng J., Ou Z., Shao Z., Di G., Yang G. (2018). CXCR2 Promotes Breast Cancer Metastasis and Chemoresistance via Suppression of AKT1 and Activation of COX2. Cancer Lett..

[76] SenGupta S., Hein L.E., Xu Y., Zhang J., Konwerski J.R., Li Y., Johnson C., Cai D., Smith J.L., Parent C.A. (2021). Triple-Negative Breast Cancer Cells Recruit Neutrophils by Secreting TGF-β and CXCR2 Ligands. Front. Immunol..

[77] Wang F., Yuan C., Wu H.-Z., Liu B., Yang Y.-F. (2021). Bioinformatics, Molecular Docking and Experiments In Vitro Analyze the Prognostic Value of CXC Chemokines in Breast Cancer. Front. Oncol..

[78] Tan J., Ge Y., Zhang M., Ding M. (2022). Proteomics Analysis Uncovers Plasminogen Activator PLAU as a Target of the STING Pathway for Suppression of Cancer Cell Migration and Invasion. J. Biol. Chem..

[79] Sarno F., Goubert D., Logie E., Rutten M.G.S., Koncz M., Deben C., Niemarkt A.E., Altucci L., Verschure P.J., Kiss A. (2022). Functional Validation of the Putative Oncogenic Activity of PLAU. Biomedicines.

[80] Pavet V., Shlyakhtina Y., He T., Ceschin D.G., Kohonen P., Perälä M., Kallioniemi O., Gronemeyer H. (2014). Plasminogen Activator Urokinase Expression Reveals TRAIL Responsiveness and Supports Fractional Survival of Cancer Cells. Cell Death Dis..

[81] Shi K., Zhou J., Li M., Yan W., Zhang J., Zhang X., Jiang L. (2024). Pan-Cancer Analysis of PLAU Indicates Its Potential Prognostic Value and Correlation with Neutrophil Infiltration in BLCA. Biochim. Biophys. Acta (BBA)—Mol. Basis Dis..

[82] Biswas S., Menon L. (2021). miRNA-Based Therapeutic Strategies. RNA-Based Mechanisms in Cancer.

[83] Zhang Z., Huang Q., Yu L., Zhu D., Li Y., Xue Z., Hua Z., Luo X., Song Z., Lu C. (2022). The Role of miRNA in Tumor Immune Escape and miRNA-Based Therapeutic Strategies. Front. Immunol..

[84] Pagoni M., Cava C., Sideris D.C., Avgeris M., Zoumpourlis V., Michalopoulos I., Drakoulis N. (2023). miRNA-Based Technologies in Cancer Therapy. J. Pers. Med..

[85] Rupaimoole R., Slack F.J. (2017). MicroRNA Therapeutics: Towards a New Era for the Management of Cancer and Other Diseases. Nat. Rev. Drug Discov..

[86] Chakraborty C., Sharma A.R., Sharma G., Doss C.G.P., Lee S.-S. (2017). Therapeutic miRNA and siRNA: Moving from Bench to Clinic as Next Generation Medicine. Mol. Ther. Nucleic Acids.

[87] Beg M.S., Brenner A.J., Sachdev J., Borad M., Kang Y.-K., Stoudemire J., Smith S., Bader A.G., Kim S., Hong D.S. (2017). Phase I Study of MRX34, a Liposomal miR-34a Mimic, Administered Twice Weekly in Patients with Advanced Solid Tumors. Investig. New Drugs.

[88] Setten R.L., Rossi J.J., Han S. (2019). The Current State and Future Directions of RNAi-Based Therapeutics. Nat. Rev. Drug Discov..

[89] Sirek T., Borawski P., Król-Jatręga K., Sirek A., Zmarzły N., Boroń D., Chalcarz M., Ossowski P., Dziobek K., Strojny D. (2025). miRNA-Mediated Regulation of Extracellular Matrix Dynamics across Breast Cancer Subtypes. Arch. Med. Sci..

[90] Sirek T., Sirek A., Zmarzły N., Opławski M., Król-Jatręga K., Boroń D., Chalcarz M., Ossowski P., Dziobek K., Strojny D. (2025). Impact of MiRNAs on Wnt-Related Gene Activity in Breast Cancer. Sci. Rep..

[91] Sirek T., Sirek A., Borawski P., Zmarzły N., Sułkowska J., Król-Jatręga K., Opławski M., Boroń D., Chalcarz M., Ossowski P. (2024). miRNAs in Signal Transduction of SMAD Proteins in Breast Cancer. Int. J. Mol. Sci..

[92] Chen Y., Wang X. (2020). miRDB: An Online Database for Prediction of Functional microRNA Targets. Nucleic Acids Res..

[93] McGeary S.E., Lin K.S., Shi C.Y., Pham T.M., Bisaria N., Kelley G.M., Bartel D.P. (2019). The Biochemical Basis of microRNA Targeting Efficacy. Science.

[94] Faul F., Erdfelder E., Lang A.-G., Buchner A. (2007). G*Power 3: A Flexible Statistical Power Analysis Program for the Social, Behavioral, and Biomedical Sciences. Behav. Res. Methods.

[95] Győrffy B. (2024). Integrated Analysis of Public Datasets for the Discovery and Validation of Survival-Associated Genes in Solid Tumors. Innovation.

[96] Győrffy B. (2024). Transcriptome-Level Discovery of Survival-Associated Biomarkers and Therapy Targets in Non-Small-Cell Lung Cancer. Br. J. Pharmacol..

